# The role of nonlinear mechanical properties of biomimetic hydrogels for organoid growth

**DOI:** 10.1063/5.0044653

**Published:** 2021-06-07

**Authors:** Benedikt Buchmann, Pablo Fernández, Andreas R. Bausch

**Affiliations:** Lehrstuhl für Biophysik E27, Physics Department and Center for Protein Assemblies CPA, Technische Universität München, 85747 Garching, Germany

## Abstract

Cell-driven plastic remodeling of the extracellular matrix (ECM) is a key regulator driving cell invasion and organoid morphogenesis in 3D. While, mostly, the linear properties are reported, the nonlinear and plastic property of the used matrix is required for these processes to occur. Here, we report on the nonlinear and plastic mechanical properties of networks derived from collagen I, Matrigel, and related hybrid gels and link their mechanical response to the underlying collagen structure. We reveal the predominantly linear behavior of Matrigel over a wide range of strains and contrast this to the highly nonlinear and plastic response of collagen upon mechanical load. We show that the mechanical nonlinear response of collagen can be gradually diminished by enriching the network stepwise with Matrigel. This tunability results from the suppression of collagen polymerization in the presence of Matrigel, resulting in a collagen network structure with significant smaller mesh size and consequent contribution to the mechanical response. Thus, the nonlinear plastic properties and structure of the ECM is not simply the addition of two independent network types but depends on the exact polymerization conditions. The understanding of this interplay is key toward an understanding of the dependencies of cellular interactions with their ECM and sheds light on the nonlinear cell-ECM interaction during organogenesis.

## I Introduction

Cell culture, regenerative medicine, and organoid assembly succeeded major progress with the establishment of 3D cell culture assays, recapitulating complex tissue architectures and developmental processes *in vivo*.^1,2^ To mimic the extracellular matrix (ECM) *in vitro,* collagen I and Matrigel have been predominantly established as reconstituted matrices.^3^ However, both matrices show distinct differences in their biochemical composition, structure, and mechanical behavior.^4–6^

Matrigel is extracted from Englebreth–Holm–Swarms mouse sarcomas and, thus, mainly consist of laminin, collagen IV, and enactin.^7^ Due to purification, Matrigel also contains various amounts of growth factors, which in turn affect cellular processes and proliferation.^8^ After polymerization, Matrigel forms a dense sponge-like network with pore sizes smaller than the common cell size.^5^ In contrast, collagen networks derived from purified collagen I solutions are reduced to a single component, resulting in a precise, biochemically controllable reconstituted matrix.^9^ Structurally, collagen exhibits a fibrillary matrix with well-defined mesh size, tunable by a broad spectrum of parameters such as crosslinker concentration, pH value, and temperature.^10,11^ Yet, such a reduced matrix is not applicable to all cell types, and especially, embryonic stem cells have been shown to proliferate preferentially in the complex milieu of Matrigel.^7,12^ Consequently, hybrid gels consisting of collagen I and Matrigel have become a tool for combining both traits,^13,14^ yet their mechanical properties are not only rather poorly characterized but also often completely ignored in biological assays.

However, besides controlling the biochemical milieu, mechanosensing of the ECM by the cells has become an additional key regulator driving 3D cell invasion.^15^ Here, mainly the stiffness of the ECM was thought to be a crucial factor in guiding the out-growth.^16,17^ Mostly in this context, stiffness is understood as the linear modulus of a gel, neglecting possible nonlinear or even plastic responses.^18,19^ Yet, recent studies have highlighted that, specifically, the nonlinear and plastic properties of the ECM are shown to sculpt the developing morphologies via pronounced fiber alignment.^20^ Explicitly, it is the plastic nature of the ECM that steers cellular migration in three-dimensional matrices.^21^ However, these nonlinear properties are becoming a major focus in materials research and still need to be fully unraveled.^22–24^ Hereby, the characteristic nonlinear strain stiffening and plastic behavior of collagen networks are of major interest.^25^ Although collagen networks polymerized at different temperatures exhibit comparable storage and loss moduli, their yield strain upon cyclic deformations differs drastically.^26^ Further, with increasing cycle number, a delay in the onset of strain stiffening can be observed. This delay is the result of single collagen fiber lengthening, buckling, and the breakage and formation of weak crosslinks.^27^ In addition, the nonlinear description gets further complicated by the fact that the observed phenomena depend on the strain amplitude, strain rate, and yield strain of the individual fibers.^28^ Mixing different types of ECM networks complicates matters; as observed for other interpenetrating network types,^29–31^ no trivial superposition can be expected. Consequently, a comprehensive mechanical characterization is of avail to provide a mechanical benchmark allowing a better comparability of different hydrogel mixtures for 3D cell cultures and to provide a frameset for the development of functional hydrogels for cell culture.

To characterize the nonlinear behavior of commonly used hydrogels for 3D cell culture, we conducted cyclic shear ramps with a constant strain rate and increasing amplitude. Hence, we highlight the nonlinear mechanical response of Matrigel and collagen I gels by comparing the cycle-dependent load and contrast the observed strain stiffening of collagen to the predominantly linear stress response of Matrigel. In addition, we reveal how the nonlinear plastic characteristics of collagen gels are reduced by the addition of Matrigel, which we link to the suppressed collagen network formation within such hybrid gels. Conclusively, it becomes apparent that collagen–Matrigel hybrid gels cannot be described solely as the sum of its components but need to be analyzed individually regarding their linear, nonlinear, and plastic properties.

## II Methods

### A Hydrogel preparation

The concentration of all hybrid gels used in this study is chosen in reference to commonly established protocols.^2,32,33^ Thus, collagen type I (Corning) purified from rat tail was diluted to a final concentration of 1.3mg/ml. Therefore, the pure collagen solution was brought to neutral pH 7.5 by mixing it with phosphate-buffered saline (PBS) and a neutralizing solution consisting of 550 mM Hepes dissolved in 11× PBS. Hereby, the volume of the neutralizing solution was a 10th of the collagen volume. The resulting collagen mixture was gently mixed and ultimately heated to 37 °C to initialize polymerization. To form crosslinked collagen suitable for cellular assays, the unpolymerized collagen solution was treated with ribose.^34^ Therefore, collagen was exposed to 200mM ribose in 0.5% acetic acid at 4 °C for 5 days prior to the experiment. Polymerization was induced similar to untreated collagen.

Growth factor–reduced Matrigel (Corning) was polymerized by heating the solution rapidly from 4 °C to 37 °C. To prevent unintended polymerization, Matrigel was handled at 4 °C while the tubes and pipette tips were stored on ice. All experiments were done with one lot of Matrigel with a determined protein concentration of 8.2 mg/ ml, which is defined here as 100% Matrigel concentration.

To prepare collagen and Matrigel hybrid gels, first PBS, neutralizing solution, and collagen were mixed prior to filling up the final volume with the desired amount of Matrigel. All hybrid gels had a final collagen concentration of 1.3mg/ml enriched by Matrigel of 25% or 50%.

### B Bulk rheology

Bulk rheology was performed using an Anton-Par Physica MCR 301 stress-controlled rheometer with a 50-mm cone-plate geometry. Gap size was set to 300 *μ*m, resulting in a sample volume of 750 *μ*l. To prevent unintended detachment of the gels, the plates were treated with 5 *μ*g/cm^2^ Cell-Tak Cell and Tissue Adhesive (Corning) and rinsed with water to remove unbound proteins. Prior to the measurements, the lower plate was cooled down to 4 °C to prevent unintended polymerization and first heated to 37 °C after the cone was lowered. To prevent evaporation, the chamber was sealed with 1 × PBS 5 min after the polymerization was initialized. Polymerization of the hydrogels was monitored by continuous oscillations with a strain amplitude of 1% at a frequency of 1 Hz. After the storage module G′ and loss module G″ equilibrated, linear properties were measured using frequency sweeps from 0.01 to 60 Hz with amplitudes of 1% strain. Subsequently, nonlinear properties were measured by applying a repetitive strain with increasing amplitude but constant strain rate as described in Sec. IIC.

In additional experiments, strain jumps were conducted to measure the relaxation module of Matrigel and collagen. Therefore, an instantaneous strain of 80% was applied to the sample and held for 80 min, while the relaxation was monitored until the stress within the samples equilibrated.

All experiments were repeated three times. For the analysis, the mean of all three samples was calculated and plotted. Representative curves are shown as indicated in the captions.

### C Strain rate-controlled ramp rheology

To quantify the nonlinear mechanical response of the hydrogels, cyclic shear ramp rheology with increasing strain amplitude and constant strain rate was performed. Henceforth, this protocol will be denoted as "$\dot{\gamma }$-pulses." During this protocol, the maximal strain was varied from 40% to 120% strain in steps of 20% with a strain rate set to 1%/s. Each strain amplitude was repeated four times. Rupturing of the sample was analyzed by increasing the strain up to 300%. Throughout the measurement, strain and stress were detected.

### D Quantification of dissipated work and plasticity

We quantified the plasticity of the different hydrogels by calculating the dissipative work *W_dissipated_* of each cycle. Specifically, we calculated the areas *A* under the loading and unloading curve and normalized it via $W_{dissipated}=\frac{A_{load}-A_{unload}}{A_{load}+A_{unload}}.$ Here, higher values refer to a higher plasticity of the networks, which is the sum of the viscous loss and plastic rearrangements within the network.

As an additional measure of dissipation, we calculated the strain- and cycle-dependent load. The load is computed by the difference between the peak stress of each loading curve at maximal strain and its stress at 0% strain at the start of the cycle. The load of each cycle was normalized by the load of the first cycle.

### E Calculation of the differential modulus

To examine the nonlinear stiffness, we calculated the strain and cycle differential modulus $K=\frac{\delta \sigma }{\delta \gamma }$ for the different strain-stress curves. The data were smoothed using a cubic spline interpolation, and the slope was numerically calculated using MATLAB R2020b. The strain value of the maximal differential modulus of the loading curve with 300% strain was defined as yield strain and the according stress as yield stress.

### F Fluorescent collagen labeling

To analyze the collagen network within different hydrogels, collagen was fluorescently labeled prior to polymerization.^35^ First, unpolymerized collagen was brought to pH 7 via dialysis at 4 ° C. Subsequently, collagen was incubated overnight with Atto 488 at 4 °C. Unbound dye was removed by an additional dialysis for 8 h. Finally, dialysis with acid was conducted overnight to prevent unintended polymerization. To prepare fluorescent collagen networks, fluorescent collagen was mixed with unlabeled collagen in a mixing ratio of 1:5. Polymerization was induced as described in Sec. II A, except hydrogels were prepared in four wells (ibidi). Pore size analysis was performed using the DiameterJ plugin developed for ImageJ.^36^

## III Results

### A Collagen structure

**Fig. 1:**
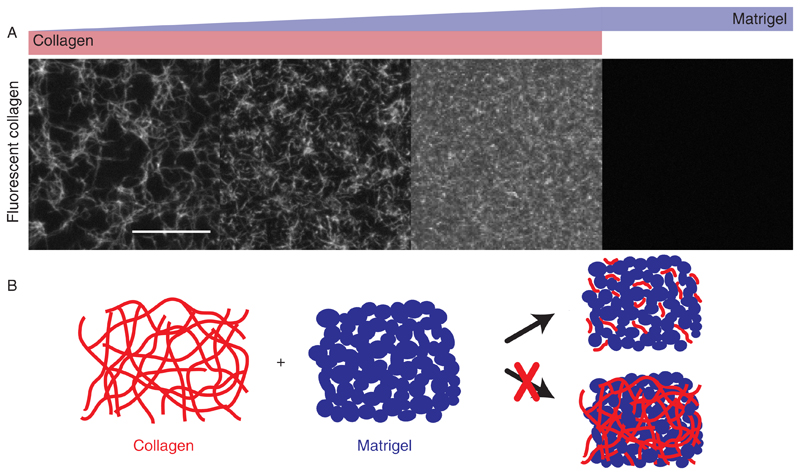
Collagen structure within different hydrogels. (a) Pure collagen (left) exhibits a fibrillary matrix with defined mesh size. Collagen enriched with 25% Matrigel (middle left) exhibits a smaller mesh size, which reduces further in collagen networks enriched with 50% Matrigel (middle right). In pure Matrigel (right), no fluorescent collagen is visible. Scale bar: 20 *μ*m. (b) Mixing of collagen and Matrigel prior to polymerization induces distinct changes in the network structure, resulting in shorter collagen fibers.

First, we analyzed the collagen network within different hydrogels using fluorescent collagen (Fig. 1 and Fig. S1). Here, pure collagen networks with a collagen concentration of 1.3 mg/ml exhibit a fibrillary matrix with a mean pore size of ~38 *μ*m^2^. Individual collagen fibers entangle with several other fibers throughout the network. By enriching collagen with 25% Matrigel, the mean pore size reduces to 23 *μ*m^2^, while individual and entangled collagen fibers are still visible. Within hybrid gels consisting of 1.3 mg/ml collagen and 50% Matrigel, the mean mesh size further reduces to 10 *μ*m^2^, with distinctly shorter fibers. The stepwise reduction of collagen mesh size with the concomitant reduction of fiber length is the consequence of the faster polymerization dynamics of Matrigel, suppressing collagen polymerization (Fig. S2).

### B Linear properties

**Fig. 2:**
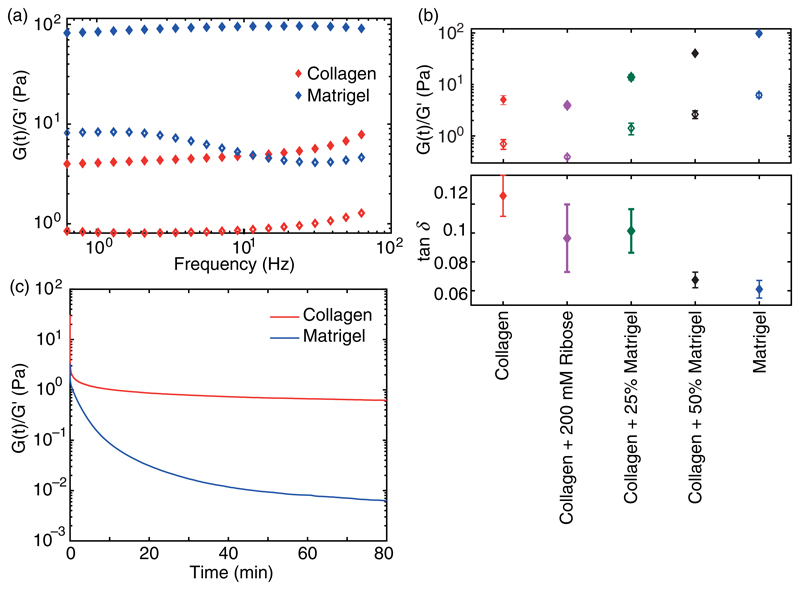
Linear properties of treated and untreated collagen gels: Matrigel and hybrid gels. (a) Storage module G′ (filled symbols) of collagen and Matrigel dominates corresponding loss module G″ independent of the frequency. (b) Comparison of G′, G″, and phase angle tan *δ* of all studied hydrogels. (c) Representative development of the relaxation module G(t) of collagen and Matrigel normalized by G′ during a strain jump of 80%.

To compare the linear mechanical properties, we measured the storage module G′ and loss module G″ by performing frequency sweeps to characterize the linear mechanical properties of collagen, Matrigel, and collagen-Matrigel hybrid gels [Fig. 2(a) and Fig. S3]. In all studied hydrogels, G′ dominated G″ independent of the frequency, highlighting the gel-like properties of each network. However, the absolute values of G′ and G″ differ drastically, indicating the different capabilities to store energy upon linear deformation [Fig. 2(b)]. In particular, pure collagen gels (1.3 mg/ml) exhibit the lowest moduli of all gels, which increases stepwise for hybrid gels with increasing amounts of Matrigel (25% and 50%). Pure Matrigel exhibits the largest G′ of 97.6 Pa compared to 4.9 Pa for pure collagen.

Further, the phase angle tand of the various hydrogels reveals a differing viscous behavior [Fig. 2(b)]. The wider phase angle of collagen highlights the higher fluidity of collagen compared to Matrigel. Through crosslinking of collagen gels or enrichment of pure collagen with Matrigel, the viscose contribution to the response function can be gradually increased.

According to the different phase angle, strain jump experiments of collagen and Matrigel reveal different relaxation behaviors. While Matrigel slowly equilibrates to zero stress, collagen relaxes nearly instantaneously but approaches a residual stress [Fig. 2(c)].

### C Nonlinear behavior

**Fig. 3:**
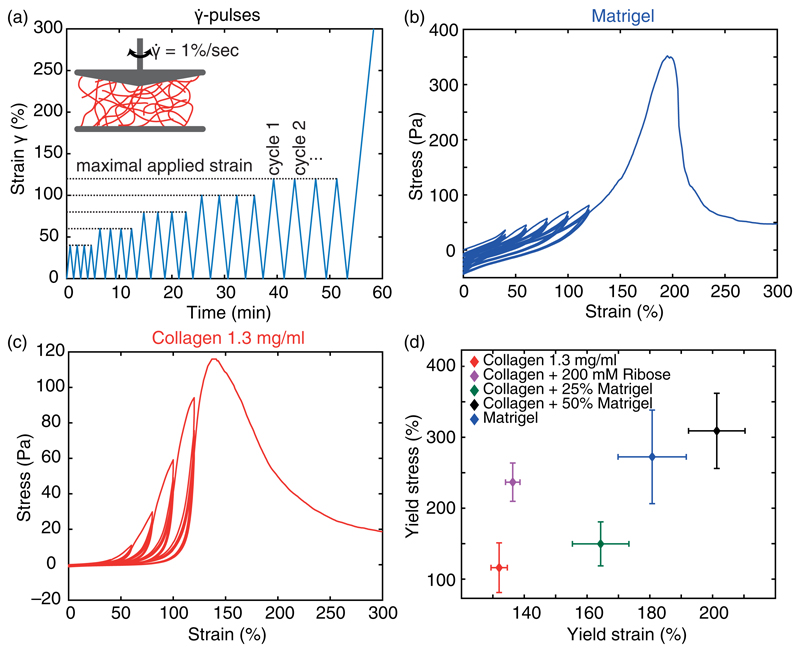
Nonlinear mechanical characteristics of the studied hydrogels. (a) Experimental protocol for bulk rheology. The inset highlights the constant strain rate $\dot{\gamma }$. (b) and (c) Representative strain—stress relation for Matrigel (b) and collagen (c) reveals different nonlinear properties. (d) Yield strain and stress for all studied hydrogels after $\dot{\gamma }$-pulses.

To probe the nonlinear behavior of the different hydrogels, we conducted a constant strain rate experiment ($\dot{\gamma }$-pulses) and measured the resulting strain-stress relation [Fig. 3(a)]. The response of Matrigel exhibits a predominantly linear response. Only at the end of each strain ramp can a slightly steeper increase in the stress be observed, resembling a nonlinear response [Fig. 3(b)]. Loading and unloading curves are just slightly shifted, resulting in an increasing offset of the stress at 0% strain, which we attribute to a plastic-viscous loss. However, only marginal differences can be observed between each pulse. In comparison, the nonlinear response of collagen is strongly pronounced, which is expressed by a distinct strain stiffening >20% strain [Fig. 3(c)]. With increasing cycle number, the onset of this strain stiffening is shifted to higher strains, resulting in an effective softening for lower strains similar to the Mullins effect.^37^ This characteristic behavior is conserved in collagen networks crosslinked via ribose [Fig. S4(a)]. Hybrid gels of collagen and 25% Matrigel exhibit characteristics of both components. Here, a diminished strain stiffening and Mullins softening can be observed. Addition of 50% Matrigel to the collagen amplifies the impact of the dominant linear mechanical response of Matrigel, with little resemblance of the mechanical response of pure collagen [Fig. S4(b) and S4(c)]. The observed response functions of the studied hydrogels are also conserved at higher concentrations (Fig. S5).

Of all studied hydrogels, collagen yields at the lowest strain and stress [Fig. 3(d)]. In comparison, the crosslinked collagen network exhibits only a slight increase in the yield strain but a more than doubled increase in the yield stress. When enriching collagen with increasing amounts of Matrigel, a stepwise increase in the yield strain can be achieved. While the addition of 25% Matrigel results in a slightly higher yield stress, 50% Matrigel exhibits a nearly threefold increase compared to pure collagen and shows the highest reachable yield strain and stress of all hydrogels. Pure Matrigel reaches a yield strain that is between the two different hybrid gels but yields at a comparable yield stress as the hybrid gels consisting of collagen and 50% Matrigel.

These properties highlight the dominance of the earlier onset of irreversible deformations in collagen networks than in Matrigel. In addition, these results emphasize the loss of collagen network structure in hybrid gels, resulting in a gradual tuning of the mechanical properties.

### D Dissipated Work

**Fig. 4:**
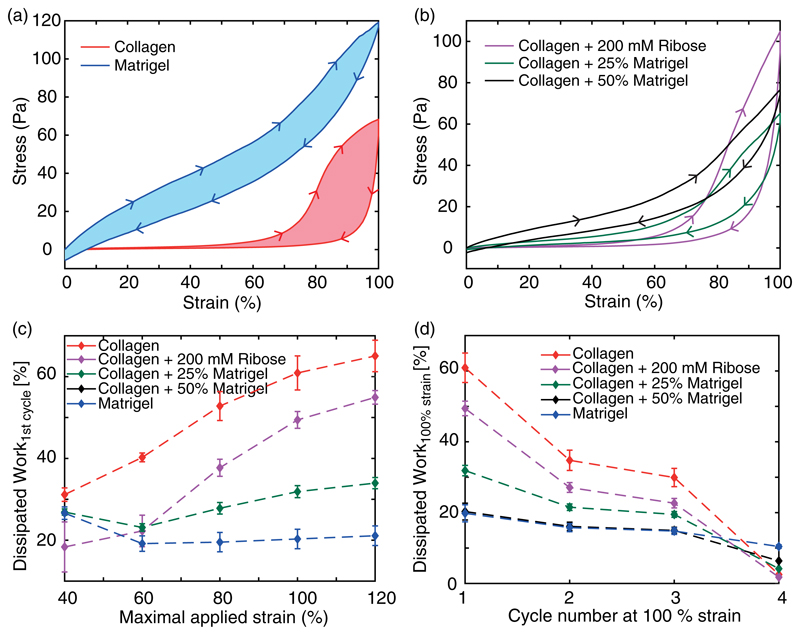
Dissipated work in loading and unloading curves. (a) and (b) Representative loading and unloading curves for the first cycle at 100% strain for collagen and Matrigel and all studied hybrid gels. Here, the filled area between the loading and unloading curves refer to the calculated dissipated work. (c) and (d) Development of the dissipated work of the first cycle against the maximal strain (c) and against the cycle number at 100% strain (d) exhibits the lower dissipated work in gels with increasing Matrigel concentration.

To characterize the plasticity of the different hydrogels, we calculated the strain- and cycle-dependent dissipated work within the loading and unloading curves [Figs. 4(a) and 4(b)]. The so-determined dissipated work hereby comprises, in general, a combination of viscous loss and dissipated energy due to plastic rearrangements.^24^ While at low strains all studied hydrogels show a similar dissipation within the first cycle, an increase in strain amplitude highlights the higher plastic properties of collagen and collagen-enriched hydrogels [Fig. 4(c)]. The dissipation within pure collagen steadily rises with strain amplitude and ultimately leads to a dissipation of up to 60% of the initial work. This behavior can be observed to be weakened in collagen gels treated with ribose. In contrast, Matrigel shows a lower and uniform dissipation throughout all strain amplitudes. This behavior is identically recapitulated by collagen gels mixed with 50% Matrigel, highlighting the dominant impact of Matrigel at this stoichiometry. Collagen gels enriched with only 25% Matrigel recapitulate the increase in dissipated work similar to pure collagen, yet the observed increase is noticeably reduced by a factor of up to two.

To further scrutinize the plastic behavior, we calculated the cycle-dependent dissipation at 100% strain [Fig. 4(d)]. Treated and untreated collagen exhibit a steadily decreasing amount of dissipative work, highlighting the reduced remodeling of the collagen network with increasing cycle number.^27^ Similar to the strain-dependent behavior, Matrigel and hybrid gels consisting of collagen and 50% Matrigel show an identical behavior with a smaller and approximately constant dissipation throughout all strain cycles. Collagen enriched with 25% Matrigel displays the attenuated characteristics of pure collagen.

### E Degree of plasticity

**Fig. 5:**
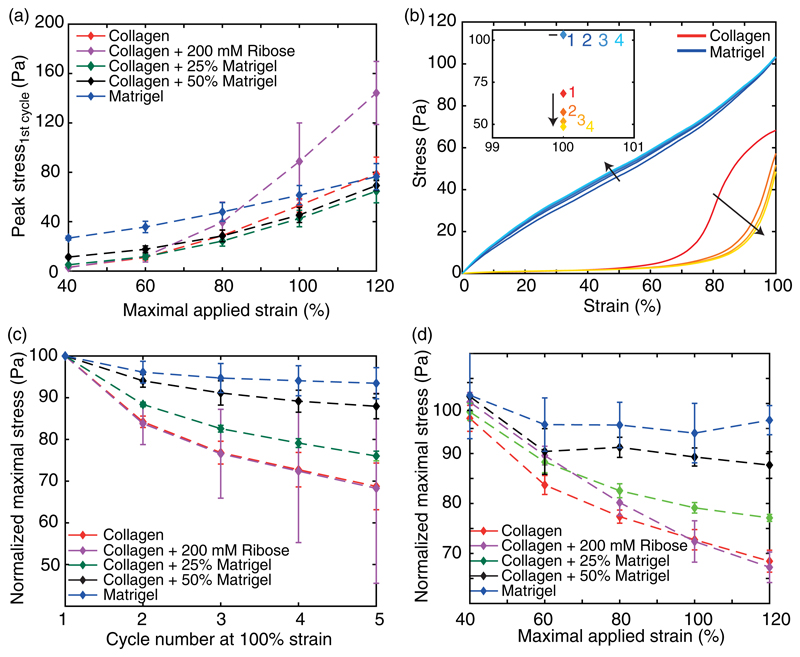
Strain- and cycle-dependent maximal load. (a) Development of the peak stress with increasing maximal strain for all studied hydrogels. (b) Representative corrected loading curves of collagen and Matrigel at 100% strain. Here, the cycle number of the loading curves is indicated by the color of the curve. While the dark red and blue correspond to the first cycle, the saturation decreases with increasing cycle number. The inset highlights the corresponding cycle-dependent development of the peak stress at 100% strain. (c) and (d) The normalized maximally reached stress depending on cycle number at 100% strain (c) and the maximal strain of the first cycle (d).

We first calculated the strain-dependent peak stress of the hydrogels. At low-strain amplitudes, collagen reaches a 10 times lower peak stress than Matrigel, which gradually increased with the stepwise addition of Matrigel [Fig. 5(a)]. However, with increasing strain amplitude and concomitant nonlinear deformations, all studied hydrogels show a steady increase in the peak stress, resulting in a congruent stress at 120% strain. Only additional crosslinked collagen reaches an up to twofold higher peak stress than the other hydrogels.

To analyze the plastic behavior of the hydrogels, we calculated the strain- and cycle-dependent load [Fig. 5(b)]. As a result, we observed the maximal plasticity of each hydrogel to be dependent on the applied strain [Fig. 5(c)]. For low-strain amplitudes, only small differences between the loading curves of each hydrogel can be observed. However, with increasing strain amplitude, the difference between the various gels becomes apparent. While treated and untreated collagens exhibit the highest plastic effect with a reduction of the peak stress of up to 30%, the stepwise addition of Matrigel gradually diminishes this effect. The addition of 25% Matrigel leads to a diminished reduction of the peak stress but conserves the strain-dependent reduction similar to collagen [Fig. 5(c)]. The addition of 50% Matrigel leads to a constant reduction of the peak stress upon strains >60%. This observed plateau highly mimics the behavior of pure Matrigel, which shows a plastic reduction of only 5%.

To further unravel the reduction of the peak stress, we highlighted the cycle-dependent plasticity at 100% strain [Fig. 5(d)]. Within five cycles, treated and untreated collagens show the highest reduction during the second cycle, with a drop of already half of the overall reduction, while the following cycles show a diminished, but persistent reduction. By the addition of 25% Matrigel, this steady decrease is conserved, but the cumulative reduction is impaired. A further increase in Matrigel concentration to 50% shows a minor reduction within the second cycle. In addition, further strain cycles only reduce the maximal load slightly, highlighting the suppressed plastic properties of the underlying collagen network and the dominating impact of Matrigel. Such a developing plateau is significant for the mechanical response of pure Matrigel. Here, the major reduction can be observed during the second cycle, which is followed by only small changes throughout further cycles. However, the maximal load only reduces by 5% of the initial value and is thereby significantly lower than the reduction observed for collagen.

Both the cycle- and strain-dependent reduction of the load highlight that the higher plasticity of collagen compared to Matrigel can be gradually changed by mixing both components in a varying stoichiometry. Remarkably, the crosslinking of collagen via ribose only shows higher reachable peak stresses yet still the same plastic behavior.

### F Differential modulus

**Fig. 6:**
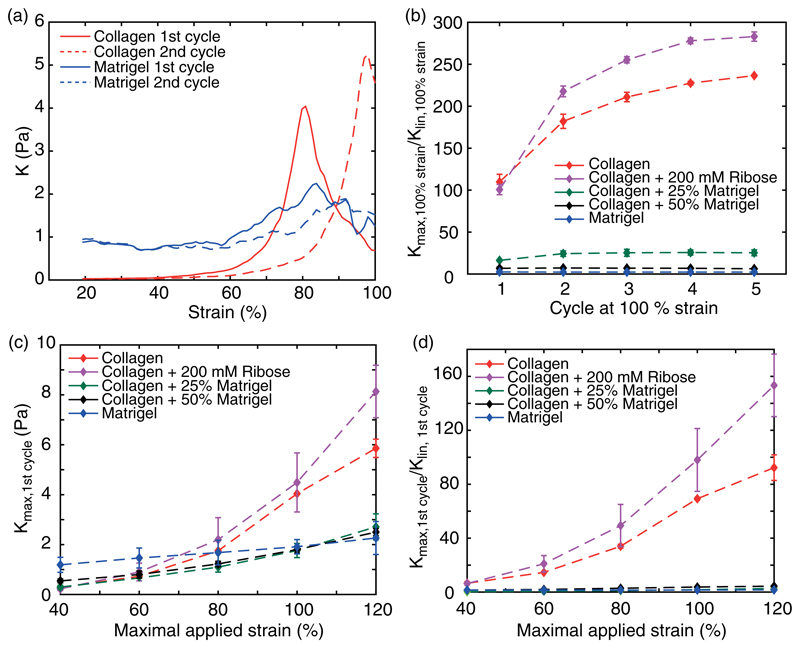
Development of the nonlinear stiffness measured by the differential modulus. (a) Representative strain dependency of the differential modulus of collagen and Matrigel at 100% strain highlights the strain stiffening for larger strains. (b) Cycle dependency of the normalized differential modulus. (c) and (d) Development of the absolute differential modulus (c) and the normalized differential modulus (d) against the maximal strain highlights the increased stiffness of collagen compared to Matrigel.

The raw loading and unloading curves of collagen already highlight the characteristic strain stiffening. To further quantify this effect, we calculated the strain- and cycle-dependent differential modulus *K* [Figs. 6(a) and 6(b) and Fig. S6]. During the first loading curve, the differential modulus of collagen slowly increases for small strains followed by a steeper increase for larger strains, indicating a strain stiffening of the network. During the second loading, the onset of increase in stiffness is shifted to higher strains, leading to a strain softening for small strain amplitudes that resembles the Mullins effect. However, this softening is followed by an increased differential modulus, highlighting its stiffer response for larger strains. In contrast, Matrigel exhibits a nearly constant stiffness during the first loading cycle only, with a minor increase at larger strains, highlighting its diminished nonlinear properties [Figs. 6(a) and 6(b)]. During the second cycle, though, the increase in *K* is slightly damped and shifted to higher strains. Likewise, hybrid gels of collagen and Matrigel exhibit a steady differential modulus throughout all cycles [Fig. 5(b)].

We observed the value of the differential modulus of all hydrogels to increase with increasing strain [Fig. 6(c)]. While at small strains *K* is comparable for all gels, pure collagen shows a distinct increase for strains >80%, ultimately leading to a two times higher *K* than Matrigel, which can be even higher in the presence of additional crosslinks. Compared to its linear response, the stiffness of pure collagen increases 100-fold and even 170-fold in the crosslinked case [Fig. 6(d)]. In contrast, the normalized stiffness for Matrigel just increases by a factor of two. However, hybrid gels of collagen and Matrigel only show a minor increase in the absolute value of K. In particular, gels composed of collagen and 25% Matrigel show a diminished 20-fold increase in *K* compared to the linear regime, underlining the highly suppressed strain stiffening of collagen by addition of Matrigel.

### G Mechanical plasticity in organogenesis

In a three-dimensional environment, cell migration is steered by the nonlinear and plastic properties of the ECM.^25,41^ During invasion, cells degrade their surrounding matrix by the use of proteases and by mechanical deformations of the matrix.^38^ The exact invasion mode is defined by the degree of matrix plasticity. While cells embedded in hydrogels with a low mechanical plasticity remodel their environment biochemically as well as mechanically, cells cultivated in hydrogels with a high plasticity exhibit a protease-independent migration mode, which solely relies on mechanical deformations.^21^ During matrix invasion, individual cells plastically align fibers in their surroundings, which then further guide the ongoing matrix invasion and enable long-ranging cell–cell interactions.^15,39^

During organogenesis, the cell-driven matrix remodeling can be further intensified but is highly dependent on the choice of extracellular matrix.^40^ Organoids grown in pure collagen gels exhibit an invasive phenotype and form filopodia-like protrusions at their invasion site.^2^ Their growth is accompanied by long-ranging, anisotropic, and non-linear ECM deformations, which cause a distinct plastic collagen fiber alignment.^41^ In comparison, the growth of organoids cultivated in Matrigel exhibits reduced ECM deformations, reveals a smooth cell-ECM boundary, and is based on local cell rearrangements within the organoid.^42^ By the use of collagen–Matrigel hybrid gels, the invasive behavior can be modulated. With increasing collagen amount, an increased invasiveness is observable,^32,43^ yet the role of mechanical plasticity is still to be determined.

Organogenesis is orchestrated by the complex interplay of bio-chemical and mechanical signaling pathways. Hereby, the occurring strains are way beyond the linear mechanical response regime. Thus, it is the nonlinear and possibly plastic mechanical responses of the surrounding matrix that dictate and steer organoid growth processes and their resulting morphology.

## IV Conclusion

In this report, we contrast the opposing linear and nonlinear mechanical properties of Matrigel and collagen and emphasize the gradually changing nonlinear behavior of hybrid gels consisting of both components with varying stoichiometry. These hybrid gels are often used in cell and organoid cultivation, yet without considering the mechanical major differences. While collagen shows a highly nonlinear and plastic behavior, Matrigel lacks these properties and exhibits an almost purely linear mechanical response without a striking plasticity. This absence of plasticity in Matrigel denies the plastic remodeling by cells, which is prerequisite for invasive cell migration and branching morphogenesis. In comparison, the plastic response of pure and cross-linked collagen with its concomitant strain softening can be described by the Mullins effect.^44^

Further, we show that by a varying mixing ratio of collagen and Matrigel, plasticity and nonlinear stiffness can be gradually engineered. While at low Matrigel concentration the hybrid gels exhibit the diminished characteristic plastic and nonlinear behavior of collagen, an increase in Matrigel leads to an increasing linear stress response and markedly reduced plasticity. Already the mixtures with 25% Matrigel show a significantly impeded plastic response. This reduction can be linked to the underlying network structure of collagen within the hybrid gels. While at high Matrigel concentrations the formation of an entangled collagen network is inhibited, low Matrigel concentrations still enable the collagen to polymerize into a fibrillary matrix but with a significantly smaller mesh size compared to pure collagen networks. In such networks, plastic remodeling processes, such as fiber alignment as well as rupture and formation of new crosslinks, are possible but to a significantly less extent.^27,28^

Previous studies have highlighted the biochemical and structural differences between collagen and Matrigel.^5,7^ Our findings introduce a rheological perspective by shedding light onto the difference in the nonlinear and plastic mechanical responses of both types of matrices. Although often only the linear moduli are reported, it is the plastic and nonlinear mechanical response that dominates the complex interaction of cells with their ECM. We have demonstrated that a dilution of collagen with Matrigel does not result in a simple combination of both network properties but leads to a severe alteration of the collagen network structure. This in turn results in drastic changes of the nonlinear plastic mechanical properties of such mixtures, which need to be taken into account. Any application based on ECM model systems for organoid growth need to take this mechanical complexity into account in order to fully address mechano-feedback processes during organogenesis.

## Supplementary Material

See the supplementary material for the detailed pore size analysis, the polymerization dynamics, and the linear and nonlinear loading curves of all studied hydrogels.

Supplementary material

Supporting information

## Data Availability

The data that support the findings of this study are available from the corresponding author upon reasonable request.
