# Supplementary material for: The role of nonlinear mechanical properties of biomimetic hydrogels for organoid growth
Source: Biophys Rev (Melville). Author manuscript; Available in PMC 2022 Jun 16. (PMC7612859; doi:10.1063/5.0044653)
Supplement: Supplementary file 1 [file EMS145498-supplement-Supplementary_material.docx]

**Supplementary Material**

**Fig. S1 Pore size analysis.** (A-C) Pore sizes distribution for (A) pure collagen, (B) collagen enriched with 25 % Matrigel and (C) collagen enriched with 50 % Matrigel.

**Fig. S2 Polymerization dynamics.** Polymerization of the hydrogels is induced by heating the samples from 4 °C to 37 °C. Hereby, the storage module of pure Matrigel and collagen equilibrates significantly faster than in hybrid gels. 

 **Fig. S3 Frequency sweeps of hybrid gels.** Storage and loss module of crosslinked collagen and hybrid gels of collagen and Matrigel in dependency of the frequency. 

**Fig. S4 Strain-stress relation of hybrid gels.** (A-C) Representative nonlinear strain-stress relation for (A) crosslinked collagen, (B) collagen enriched with 25 % Matrigel and (C) collagen enriched with 50 % Matrigel.

**Fig. S5 Concentration-dependent nonlinear response function.** (A) Collagen at a concentration of 2 mg/ml exhibits the same cyclic strain-stiffening and Mullins-softening as at lower concentrations. (B) Matrigel reveals a predominantly linear strain-stress relation independent on the concentration. Yet, yield strain and stress changes in dependency on the concentration. (C-D) Hybrid gels with a collagen concentration of 2 mg/ml show the same impaired nonlinear mechanical response with increasing Matrigel concentrations similar to hybrid gels with a collagen concentration of 1.3 mg/ml.

**Fig. S6 Differential module of hybrid gels.** Cycle-dependent differential module of crosslinked collagen and hybrid gels of collagen and Matrigel at 100 % strain.
