# Supplementary material for: The role of nonlinear mechanical properties of biomimetic hydrogels for organoid growth
Source: Biophys Rev (Melville). Author manuscript; Available in PMC 2022 Jun 16. (PMC7612859; doi:10.1063/5.0044653)
Supplement: Supplementary file 1 [file EMS145498-supplement-Supporting_information.pdf]

Supporting information

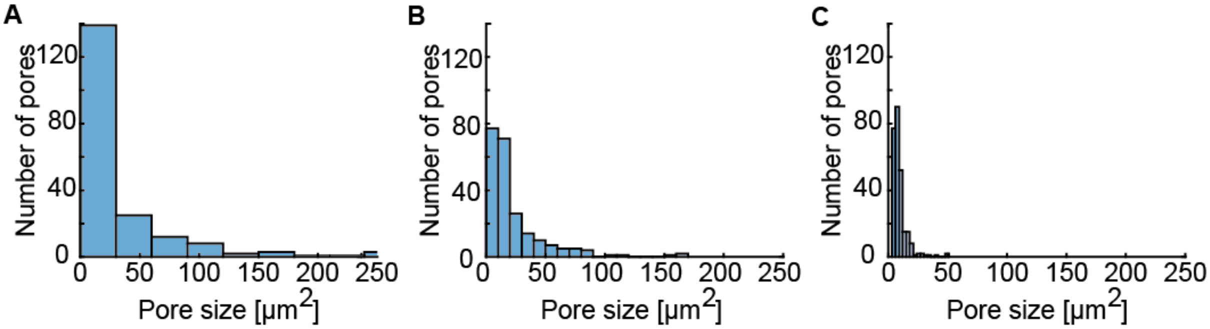

Fig. S1 Pore size analysis. (A-C) Pore sizes distribution for (A) pure collagen, (B) collagen enriched with 25 % Matrigel and (C) collagen enriched with 50 % Matrigel.

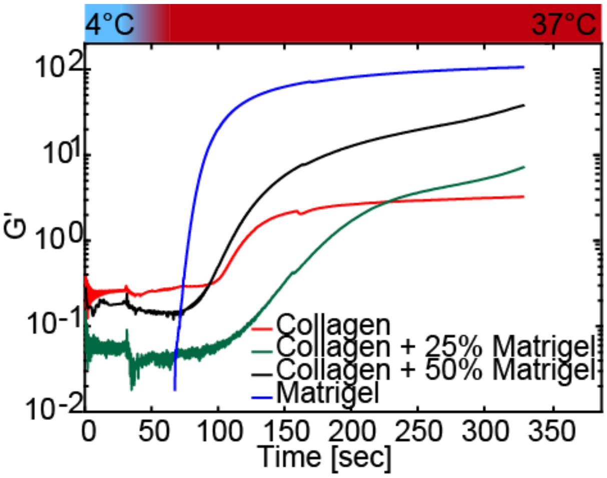

Fig. S2 Polymerization dynamics. Polymerization of the hydrogels is induced by heating the samples from 4 °C to 37 °C. Hereby, the storage module of pure Matrigel and collagen equilibrates significantly faster than in hybrid gels.

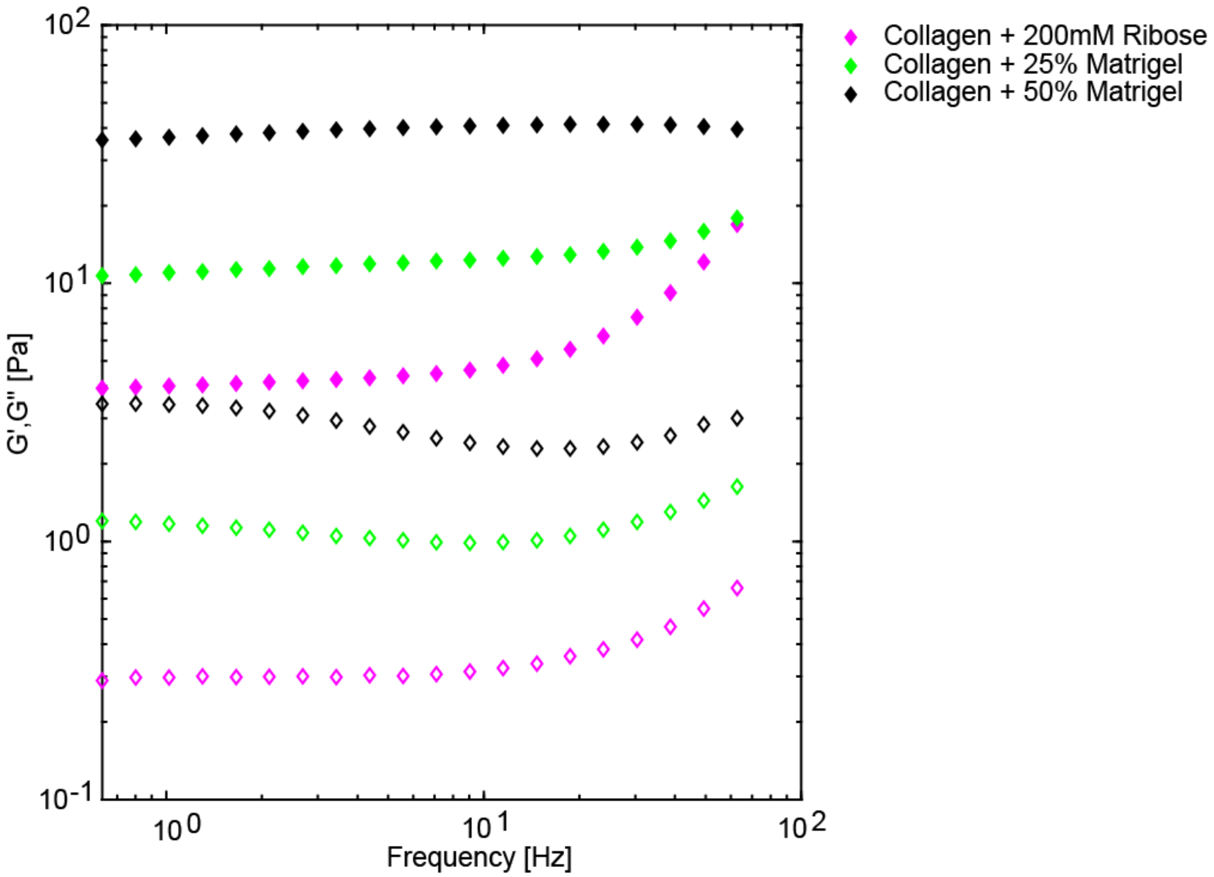

Fig. S3 Frequency sweeps of hybrid gels. Storage and loss module of crosslinked collagen and hybrid gels of collagen and Matrigel in dependency of the frequency.

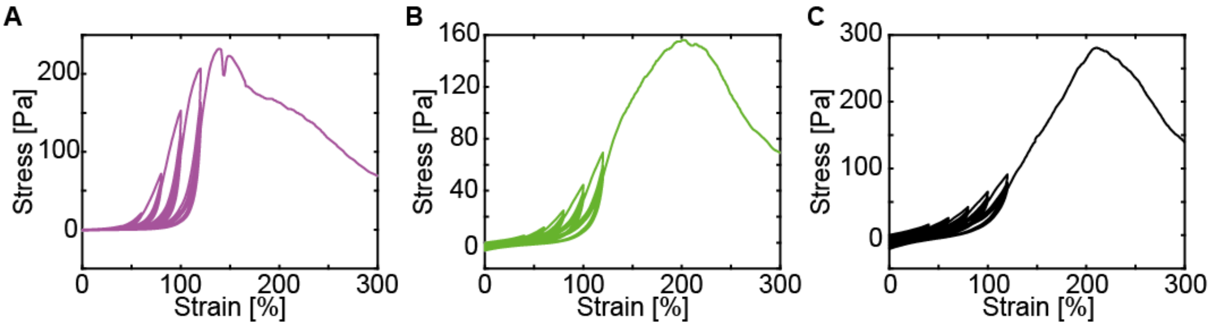

Fig. S4 Strain-stress relation of hybrid gels. (A-C) Representative nonlinear strain-stress relation for (A) crosslinked collagen, (B) collagen enriched with 25 % Matrigel and (C) collagen enriched with 50 % Matrigel.

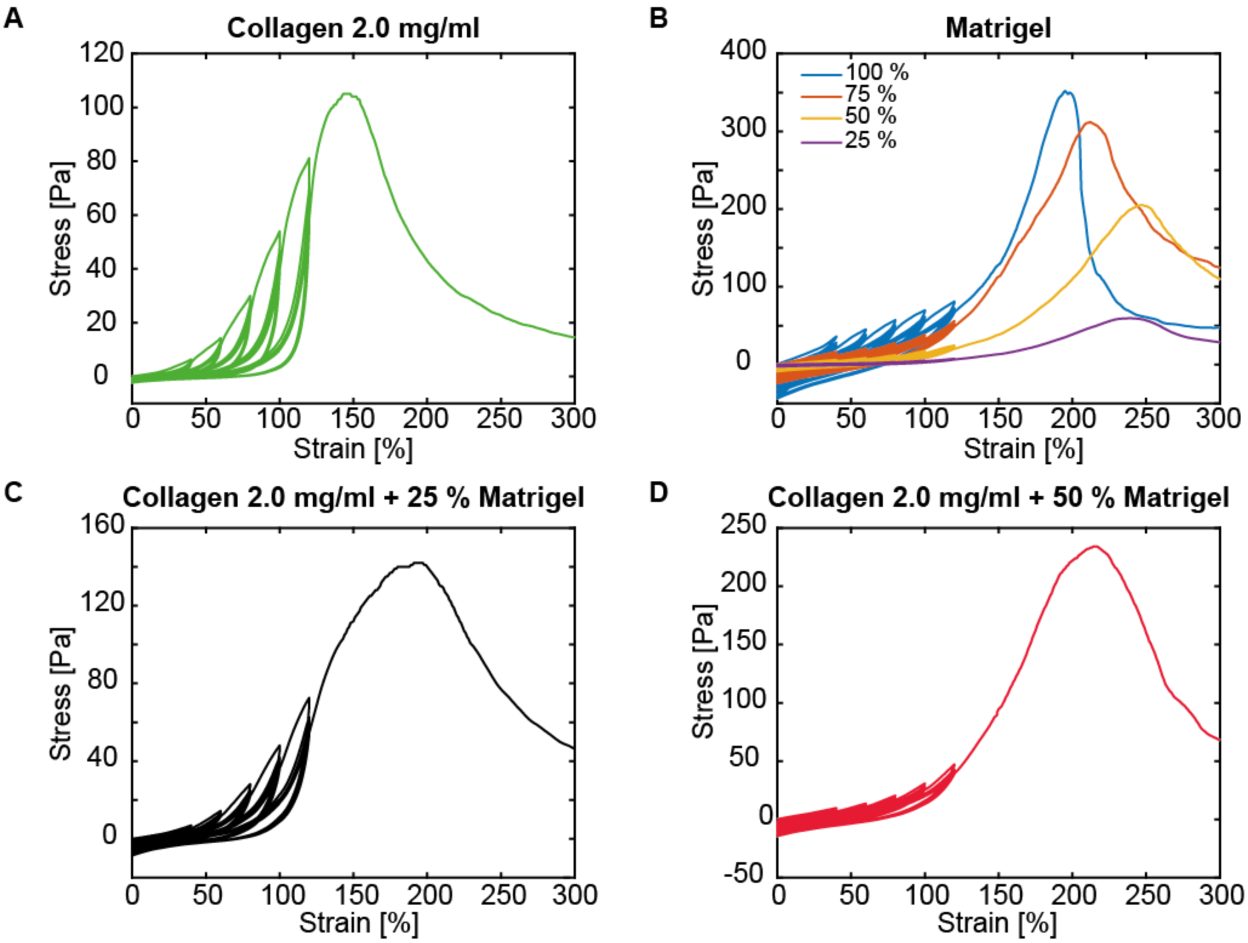

Fig. S5 Concentration-dependent nonlinear response function. (A) Collagen at a concentration of 2 mg/ml exhibits the same cyclic strain-stiffening and Mullins-softening as at lower concentrations. (B) Matrigel reveals a predominantly linear strain-stress relation independent on the concentration. Yet, yield strain and stress changes in dependency on the concentration. (C-D) Hybrid gels with a collagen concentration of 2 mg/ml show the same impaired nonlinear mechanical response with increasing Matrigel concentrations similar to hybrid gels with a collagen concentration of 1.3 mg/ml.

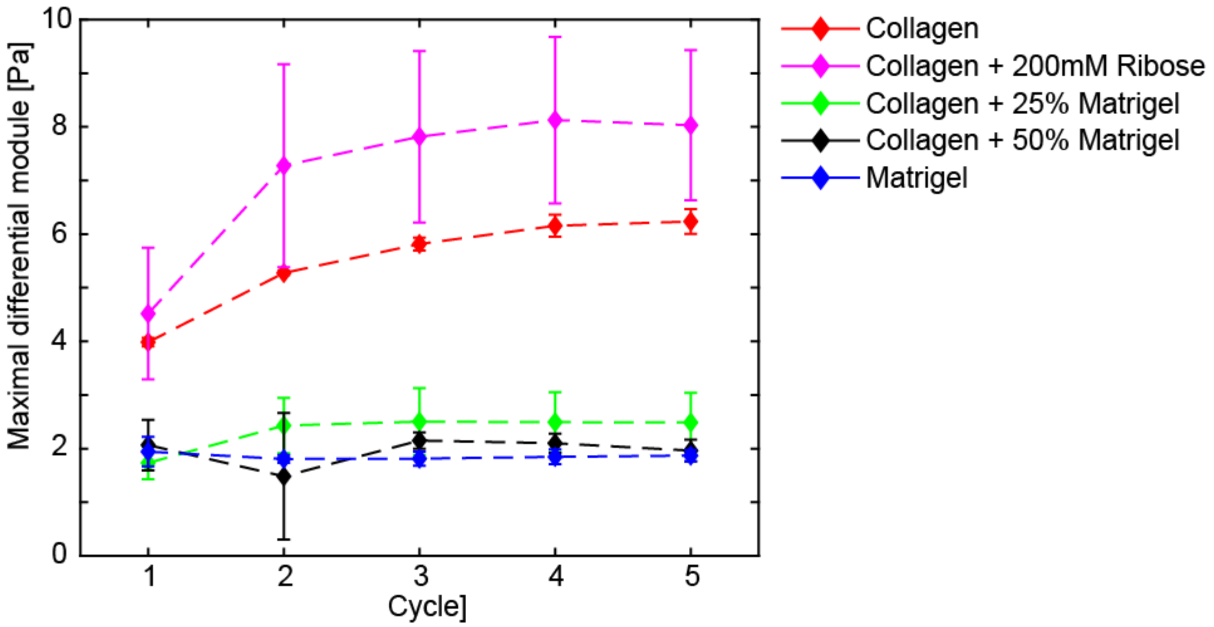

Fig. S6 Differential module of hybrid gels. Cycle-dependent differential module of crosslinked collagen and hybrid gels of collagen and Matrigel at 100 % strain.
